# Genetic Heterogeneity of Familial Hypercholesterolemia: Repercussions for Molecular Diagnosis

**DOI:** 10.3390/ijms24043224

**Published:** 2023-02-06

**Authors:** Maria Donata Di Taranto, Giuliana Fortunato

**Affiliations:** 1 Dipartimento di Medicina Molecolare e Biotecnologie Mediche, Università degli Studi di Napoli Federico II, 80131 Naples, Italy; 2 CEINGE-Biotecnologie avanzate Franco Salvatore, 80145 Naples, Italy

**Keywords:** familial hypercholesterolemia, genetics, FH phenocopies, oligogenic FH, polygenic risk score, modifier factor, genetic heterogeneity, phenotypic variability, molecular diagnosis

## Abstract

Genetics of Familial Hypercholesterolemia (FH) is ascribable to pathogenic variants in genes encoding proteins leading to an impaired LDL uptake by the LDL receptor (LDLR). Two forms of the disease are possible, heterozygous (HeFH) and homozygous (HoFH), caused by one or two pathogenic variants, respectively, in the three main genes that are responsible for the autosomal dominant disease: *LDLR*, *APOB* and *PCSK9* genes. The HeFH is the most common genetic disease in humans, being the prevalence about 1:300. Variants in the *LDLRAP1* gene causes FH with a recessive inheritance and a specific *APOE* variant was described as causative of FH, contributing to increase FH genetic heterogeneity. In addition, variants in genes causing other dyslipidemias showing phenotypes overlapping with FH may mimic FH in patients without causative variants (FH-phenocopies; *ABCG5*, *ABCG8*, *CYP27A1* and *LIPA* genes) or act as phenotype modifiers in patients with a pathogenic variant in a causative gene. The presence of several common variants was also considered a genetic basis of FH and several polygenic risk scores (PRS) have been described. The presence of a variant in modifier genes or high PRS in HeFH further exacerbates the phenotype, partially justifying its variability among patients. This review aims to report the updates on the genetic and molecular bases of FH with their implication for molecular diagnosis.

## 1. Introduction

Familial Hypercholesterolemia (FH) is a very heterogeneous genetic disease leading to high plasma low-density lipoproteins (LDL) cholesterol levels and premature atherosclerosis-based cardiovascular diseases (ASCVD) [1,2]. The FH heterogeneity is related both to the heterogenous genetic basis and to the very variable phenotype observed among affected patients.

Molecular bases of FH are related to an impaired LDL uptake by the LDL receptor (LDLR), a condition genetically inherited mainly through pathogenic variants in the genes encoding for LDLR (*LDLR* gene), apolipoprotein B (ApoB—*APOB* gene) and Proprotein Convertase Subtilisin/Kexin Type 9 (*PCSK9* gene) [1]. FH is an autosomal dominant disease that can be either heterozygous (HeFH) or homozygous (HoFH), depending on the number of alleles carrying a pathogenic variant [2].

HeFH is the most common genetic disease in humans. A prevalence of 1:250 in the general population was estimated by a meta-analysis in 2017 [3], whereas more recently, it was estimated at 1:311 and 1:313 by two independent meta-analyses on 7,297,363 and 10,921,310 subjects, respectively [4,5]. However, according to the latter study, in 90% of world’s countries the national prevalence of FH is unknown. The FH prevalence is especially high in specific groups of patients, such as those with ASCVD (1:17), according to Hu et al. [4] or those affected by premature ischemic heart disease (prevalence of 1:15) and severe hypercholesterolemia (1:14) according to Beheshti SO et al. [5]. In 2022, a meta-analysis demonstrated that FH prevalence varies across ethnicity, ranging from 1:400 in Asian individuals to 1:192 in black and brown individuals [6]. A high FH prevalence implies that most of FH patients remain undiagnosed and therefore untreated, as noted by the European Atherosclerosis Society (EAS) consensus paper already in 2013 [1]. It suggests to deeply analyze the patient characteristics and familial history in order to inquire about FH presence in case of high LDL-cholesterol levels and of familiarity for high LDL-cholesterol levels.

Since FH has a dominant inheritance, such a wide distribution of gene allele carrying a pathogenic variant appears to be quite unexpected. The high prevalence of FH-causative alleles can be justified by the fact that these alleles can be considered thrifty, i.e., genotypes with a pathogenic variant may provide a selective advantage during evolution due to the possibility to maintain high circulating cholesterol levels even during eras of food shortages [7]. In addition, no negative selection of FH alleles occurs. In fact, ASCVD development happens late in the life of FH patient, after the usual reproductive age and, therefore, the disease presence does not impair the capacity of affected patients to transmit the mutated alleles to new generations [7]. In short, during natural evolution, alleles with FH-causative variants undergo a positive selection and did not undergo a negative selection, despite the severe cardiovascular consequences.

From a simple PubMed search with the words “Familial hypercholesterolemia”, 579 results were retrieved in 2022 alone (search performed on 30 December 2022), indicating that frequent updates of literature reviews are required.

## 2. Genetics and Molecular Basis

Not all severe hypercholesterolemias are FH [8]. FH is a genetic disease in which a clear dominant inheritance should be observed within affected families. Several criteria are available to clinically identify potentially affected patients, as reported in reference [9].

Genetic bases of the autosomal dominant FH are typically ascribable to pathogenic variants in the three major genes related to LDL uptake, *LDLR*, *APOB* and *PCSK9*, with a high number of different causative variants. However, additional genetic alterations could determine or exacerbate the FH phenotype, thus further contributing to the genetic heterogeneity of the disease.

### 2.1. Classical FH-Causative Genes

In the endogenous pathway of lipid metabolism, the liver secretes triglyceride-rich lipoproteins called very low-density lipoproteins (VLDL), as lipoprotein subclasses are defined based on their density. After triglyceride hydrolysis by different lipases in turns, the VLDLs produced by the liver are progressively depleted of both triglycerides and their protein constituents (apolipoproteins), first turning into intermediate-density lipoproteins (IDL), then into LDL, requiring receptor-mediated endocytosis to be removed from blood [10]. The molecular alteration leading to FH is the impaired clearance of LDLs by their receptor, the LDLR, the only one receptor binding ApoB. This could happen in presence of a pathogenic variant in *LDLR*, *APOB*, *PCSK9* and *LDLRAP1* genes.

The *LDLR* gene is the first gene identified as causative of FH by Goldstein J. and Brown M., who were awarded the Nobel prize in Medicine in 1985 for this discovery [11]. Since then, more than 2000 rare variants in the *LDLR* gene have been reported [9].

Five functional classes define the different mechanisms underlying a dysfunctional LDLR: class 1: absence of protein synthesis; class 2: altered protein maturation and exposure on the plasma membrane; class 3: impaired binding to ApoB; class 4: impaired endocytosis; and class 5: defective recycle on the plasma membrane after endocytosis [12].

The *LDLR* variants are the most frequent cause of FH in several regions [13,14,15,16]. Despite the high number of different pathogenic variants reported in the *LDLR* gene, some of these show a very high frequency in specific geographic regions due to the presence of a founder effect [17,18].

As to ApoB, not all the dysfunctional proteins lead to a hypercholesterolemic phenotype. In fact, the *APOB* genetic variants leading to an absent protein or to a protein unable to assemble in VLDLs cause low plasma cholesterol levels due to a defective synthesis of VLDLs by liver [19]. Variants in the *APOB* gene leading to FH are those causing an impaired binding of ApoB to LDLR [20], i.e., mainly those present in the gene regions encoding for the LDLR binding domains (exons 26 and 29), even though variants present in other regions were described [21].

The *PCSK9* gene is the latest discovered gene [22], but probably the most relevant one for FH management because its discovery allowed the identification of the molecular mechanism that is mostly targeted by innovative therapies based on its inhibition by monoclonal antibodies or small interfering RNAs [23]. The encoded protein is able to decrease the number of LDLRs on the plasma membrane through different mechanisms taking place either in the extracellular region or inside the cell. After the binding to the LDL-LDLR complex, the secreted PCSK9 is internalized with it triggering LDLR degradation rather than its recycling on the cell membrane [24]. An intracellular mechanism leading to low levels of LDLR was also described, but not completely disclosed [25,26]. FH-causative variants lead to an increased PCSK9 activity (Gain-of-function—GOF), whereas those leading to a decreased PCSK9 activity (Loss-of-function—LOF) are causative of decreased LDL-cholesterol levels [24].

The *LDLRAP1* gene encoding for the protein mediating the interaction of LDLR with the clathrin-coated pits (LDLR adaptor protein 1) is responsible for autosomal recessive hypercholesterolemia (ARH), being causative of the disease only if both alleles carry a pathogenic variant. In absence of this protein, the LDL–LDLR complex cannot be internalized. ARH is an extremely rare form of genetic hypercholesterolemia showing clinical features common to the HoFH [27]. An interesting feature of the disease diffusion is that it is mainly prevalent in specific populations, due to a founder effect [27].

Extreme phenotypic variability can be observed among FH patients with the same genetic status, which was partially explained by the different impact of the mutated gene or by the different variant types, in both adult and pediatric patients [28,29,30,31,32]. The genetic status and the variant type were also associated with different responses to PCSK9 inhibitors regarding both the LDL-cholesterol lowering effect and the changes in carotid stiffness [33,34].

### 2.2. Heterozygous and Homozygous FH

**Figure 1:**
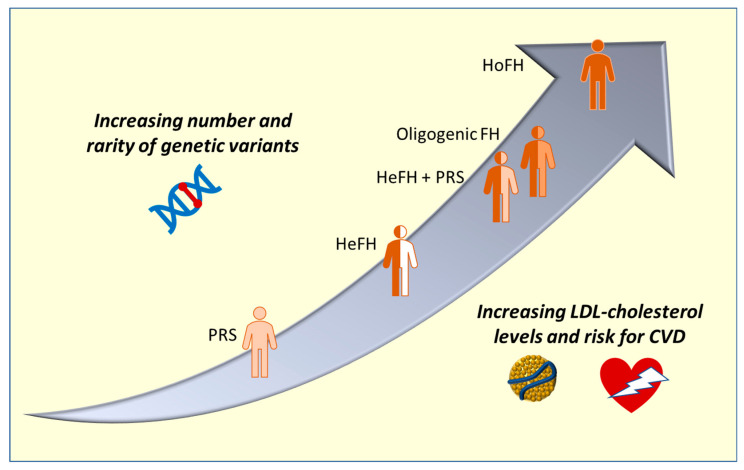
Different genetic status at the basis of hypercholesterolemia and relation with phenotype. The y-axis indicates the number of rare genetic variants, whereas the x-axis indicates the increase in LDL-cholesterol and the associated CVD risk. PRS: Polygenic risk score; HeFH: heterozygous Familial hypercholesterolemia (FH); HoFH: homozygous FH; CVD: cardiovascular disease.

Since the above three genes act on the phenotype with a co-dominant inheritance, the number of alleles carrying a pathogenic variant determines the phenotype severity being associated with the two forms of the disease, the HeFH and the HoFH. In the former, a single pathogenic variant is present in one of the three causative genes, whereas two pathogenic variants are present in the latter [2]. The term “HoFH” indicates a clinical condition characterized by very high LDL-cholesterol levels (usually 300–500 mg/dL) and very early onset of ASCVD (also in childhood if untreated) rather than a genetic state [2]. In fact, HoFH can be caused by true homozygosis (the same variant on the two alleles of a gene), compound heterozygosis (two different variants in the two alleles of a gene), and double heterozygosis (two variants at heterozygous status in two different genes); biallelic variants in the *LDLRAP1* gene can also be causative of HoFH [35] (Figure 1). The prevalence of HoFH was estimated as approximatively 1:300,000 [35]; in particular, we reported a frequency of 1:320,000 in a region of Southern Italy, although it is likely that further HoFH patients remain undetected [36].

Despite the very high LDL-cholesterol levels suggestive of FH disease, a recent study on the largest HoFH cohort, collected from 38 countries, has indicated that diagnosis is not always promptly performed [35]. Thanks to the wide use of next-generation sequencing (NGS), the identification of HoFH patients will be improved since variants in all causative genes can be detected at the same time. An accurate and comprehensive genetic screening is essential for the identification of HoFH patients allowing them to access to the most appropriate therapies that revealed to be very promising. Since in case of biallelic pathogenic variants in the *LDLR* gene, only a very low residual activity of the LDLR is present, novel and effective therapies for HoFH are independent of the LDLR activity. These therapies include lomitapide for the inhibition of microsomal triglyceride transfer protein (MTTP), leading to a decreased production of VLDLs by the liver [37,38] or monoclonal antibodies for the inhibition of the Angiopoietin-Like Protein 3 (ANGPTL3), which was proved to increase IDL and LDL clearance by LDLR independent pathways [39]. Bempedoic acid is another drug acting independently of LDLR used in HeFH patients; it is an inhibitor of the ATP citrate lyase, the enzyme transforming citrate into acetyl-CoA molecules used for fatty acid synthesis [40]. A recent paper has reviewed the new therapies for FH management in relation to the genetic status of patients [23].

### 2.3. Other Genes Involved in FH

No pathogenic variants were identified in a consistent percentage of FH patients [41,42] suggesting that additional causative genes should be identified. Unfortunately, attempts to identify new FH-causative genes ended up detecting another dyslipidemia (sitosterolemia) [43], genes or loci which were never confirmed [44,45,46] or whose role was subsequently disowned [47].

In 2013, a rare variant in the *APOE* gene leading to the deletion of leucine 167 of protein was described in a French family with FH [48], making this another FH causative gene. Additionally, an *APOE* missense variant at homozygous status was described in a child with FH; in this case the presence of the heterozygous variant was not associated with the disease [49]. For years, the ApoB was considered the only remaining apolipoprotein in LDL, whereas it was recently demonstrated that also a few molecules of Apolipoprotein E (ApoE) are present in this lipoprotein class [50].

It is clear that *APOE* variants lead to the disturbance of lipoprotein metabolism, involving both cholesterol and triglyceride lipid classes [51]. Among the others, *APOE* rare variants were associated with lipoprotein glomerulopathy and familial combined hyperlipidemia (FCH), whereas the homozygosity for the E2 polymorphic variant is associated with dysbetalipoproteinemia. The involvement of the *APOE* gene in several dyslipidemias is emblematic of the interrelation between the metabolisms of different lipid classes. A recent screening showed the involvement of several rare variants in the *APOE* gene in both autosomal dominant hypercholesterolemia and FCH [52]. Sometimes, the partial overlapping phenotype and familiarity makes it hard to distinguish FH from FCH. Genetic screening can contribute to the identification of FH-causative variants among FCH patients as previously described [53,54,55]. However, due to the rarity of each variant the biochemical analysis of several unrelated patients was not performed, and therefore it could not be proved whether the same variant can be associated with different lipid alterations.

The *STAP1* gene was initially identified as a new FH-causative gene [47], but further evidence disowned this role [56]. Other loci were candidate as potential FH-causative FH [45,46], although the genes have never been identified.

### 2.4. FH Phenocopies

The term “FH phenocopies” is widely used to indicate the genes whose pathogenic variants can induce diseases with a phenotype partially overlapping the one of FH, i.e., mainly high cholesterol and xanthomas [29,57]. It should be noted that all these diseases are inherited by an autosomal recessive trait, hence only the presence of biallelic variants (homozygosity or compound heterozygosity) may cause disease.

1. Among these genes, the *ABCG5* and *ABCG8* encoding for the two subunits of the sterol transporter mediating efflux of plant sterols both in the enterocytes, immediately after their absorption or in the liver causing their excretion in the bile [58]. These genes are causative for sitosterolemia, an autosomal recessive disease leading to severe xanthomatosis and high cholesterol levels. Variants in the *ABCG5* and *ABCG8* genes were identified in 2.4% of Dutch FH patients [59].
2. Cerebrotendinous xanthomatosis is another disease characterized by diffuse xanthomas and, in some cases, by high cholesterol levels that can mimic FH [60,61]. Variants in the causative gene, the *CYP27A1*, were identified in suspected FH patients without other pathogenic variants [62] or in presence of a FH-causative variant as worsening effects [63]. The molecular defect is due to the absence of functional sterol 27-hydroxylase, an enzyme essential for turning cholesterol in bile acids.
3. The *LIPA* gene encodes for the lysosomal acid lipase, the enzyme essential for the degradation of cholesteryl esters that in absence of a functional enzyme accumulate within lysosomes giving rise to a severe disease characterized by high cholesterol levels that can be misdiagnosed as FH (Lysosomal Acid Lipase Deficiency—LALD) [64]. In Slovenia, during the universal screening for FH, 3 children suffering from LALD were identified among hypercholesterolemic children [65].

The correct identification of patients suffering from the above diseases allows to modify the patient therapy accordingly. In fact, sitosterolemia can benefit from ezetimibe inhibiting the transported Niemann-Pick C1-Like 1 (NPC1L1), thus reducing intestinal sterol absorption, whereas cerebrotendinous xanthomatosis is usually treated by chenodeoxycholic acid, one of the molecules synthesized by sterol 27-hydroxylase. As for LALD, the replacement therapy is the most effective treatment.

### 2.5. Oligogenic FH and Modifier Genes

Oligogenic FH was first defined in 2018 as the contemporary presence of a heterozygous variant in one of the FH-causative genes and of a heterozygous variant in genes causing recessive hypercholesterolemia (*LDLRAP1*), sitosterolemia (*ABCG5* and *ABCG8*) or *APOE* [66]. However, without the use of the term “oligogenic”, these genetic conditions were previously identified [63,67]. Thanks to the analysis of multiple genes at the same time by NGS, several studies highlighted the complex involvement of multiple genes in hypercholesterolemia, worsening the FH phenotype.

Two cases of oligogenic hypercholesterolemia with variants in three genes were recently reported. A French family was examined by NGS and the segregation analysis revealed an oligogenic form of FH caused by the contemporary presence of heterozygous variants in two genes (*LRP6* and *CYP7A1* genes) further complicated by a heterozygous variant in the *LDLRAP1* gene in some family members [68]. In this case, a single variant was not associated with a FH-like phenotype that was instead present as a combined effect of 2 or more variants. In Japan, a case of FH with the contemporary presence of a potential pathogenic variant in the *PCSK9* gene, a pathogenic variant in the *ABCG5* gene and a homozygous variant in the *CD36* gene was reported [69]. CD36 is a receptor for oxidized-LDL and a transporter of fatty acids; its deficiency was caused by biallelic variants in the *CD36* gene, is associated with high LDL-cholesterol levels, worsening the FH phenotype in the patient.

If some genetic variants can exacerbate the hypercholesterolemic phenotype, it is feasible that other genetic variants are likely do the opposite. In fact, Huijgen et al. analyzed patients carrying a FH-causative variant with normal LDL-cholesterol levels and identified variants in *APOB*, *PCSK9* and *ANGPTL3* genes, potentially lowering LDL-cholesterol levels [70]. Additionally, the *MTTP* gene was investigated as a gene likely to mitigate FH phenotype [71], and the coexistence of FH and hypobetalipoproteinemia was described [72].

The possibility of multiple variants in FH causative genes one leading to high cholesterol levels and the other to low cholesterol levels highlights that during cascade screening the analysis of all causative genes may be useful for a comprehensive evaluation of the genetic basis of lipid alterations.

High circulating levels of Lipoprotein(a) can contribute to the manifestation of hypercholesterolemia mimicking the FH phenotype [73]. High Lipoprotein(a) levels are inherited because they are genetically determined by a low repetition number of the Kringle IV type 2 domain [74]. Due to genetic inheritance, high Lipoprotein(a) familiarity, similar to genetic hypercholesterolemia, can be observed. Lipoprotein(a) should be regarded as a mimicking and modifier factor of the hypercholesterolemia phenotype as recently reported by Marco-Benedí et al. [75]. A sub-study of the Italian LIPIGEN registry of genetic dyslipidemias reported that high Lipoprotein(a) levels were associated with a positive history of ASCVD in FH children [76]. The potential of including Lipoprotein(a) as an integration of the clinical FH diagnosis was recently reviewed [77].

### 2.6. Polygenic Risk Scores

The difficulty in identifying a clear genetic cause in several FH patients, suggested that the disease could be caused not only by a rare variant with a great impact on a protein function, but also by the contemporary presence of several common variants (polymorphisms) in different genes, each of which with a moderate impact on protein function. A specific weight is assigned to each polymorphism and a total score is calculated (Polygenic risk score—PRS). If the score is greater than a defined threshold, the polygenic basis of hypercholesterolemia is established. The first score described in patients with a clinical suspect of FH was built on 12 polymorphisms [78], and afterwards refined considering only 6 polymorphisms [79]. Both scores were slightly higher in FH patients without pathogenic variants than in healthy controls.

However, due to different genetic background PRS set-up in a population cannot be applied to another, making the PRS poorly generalizable [80] and suggesting creating different PRS. In addition, thanks to NGS, PRS including more and more polymorphisms were constructed with the aim to improve diagnostic ability; the most recent score was constructed on 165 polymorphism, promising to outperform the previous ones [81].

Based on a study on 16,324 subjects from different ethnicities, a larger portion of subjects with very high LDL-cholesterol levels was better explained by PRS than by monogenic causes [82], suggesting that PRS could be useful to make a diagnosis of hypercholesterolemia. The presence of a diagnosed genetic basis for hypercholesterolemia could improve patient compliance with therapy. Even in patients with a recognizable genetic cause of FH, PRS can impact the lipid profile and the risk for ASCVD, further explaining the phenotypic variability typical of FH, similarly to the case of oligogenic FH [83].

Taken together, these data indicate that PRS can be considered as both an alternative cause of hypercholesterolemia and as a modifier factor. Table 1 reports the involved genes and the different genetic conditions observed among patients with FH.

## 3. Molecular Diagnosis

Genetic analysis of FH patients can now be performed by NGS decreasing costs and time and while increasing the number of screened genes.

Certainly, the main causative genes must be thoroughly analyzed to avoid missing the diagnosis of patients carrying variants in genes with a low prevalence of pathogenic variants, but also to be able to identify compound heterozygotes and double heterozygotes. As an example, FH-causative variants in the *APOB* gene are mainly located in the exon 26 and 29, the gene regions mostly investigated in the sequencing studies conducted with traditional low throughput methods. To date, NGS allows to analyze the whole *APOB* gene, thereby identifying FH-causative variants also in the other regions [21]. Furthermore, a high coverage should be maintained to be able to analyze copy number variants (CNV) [84] accounting for a considerable percentage of *LDLR* pathogenic variants. A CNV consisting in the duplication of the whole *PCSK9* gene was also described [85].

Genes representing FH-phenocopies should be included to rule out the presence of different genetic dyslipidemias (biallelic variants) or to identify cases of oligogenic FH (a pathogenic variant in causative genes together with a pathogenic variant in other genes). Actually, due to the interrelation between different lipid traits and the ascertained involvement of genes causative of different dyslipidemias in modulating FH phenotype, the analysis of all known genes related to lipid metabolism could be useful to identify oligogenic cases. Through a widespread use of NGS complex genotypes at underlying FH could be more easily observed.

Since PRS may help to explain a large portion of hypercholesterolemic patients, albeit not FH, it should be evaluated when no clear pathogenic variants were reported. The presence of a PRS associated with hypercholesterolemia could improve patient compliance.

As to lipoprotein(a), several single nucleotide polymorphisms in the *LPA* gene were identified as being associated with a low number of Kringle IV type 2 domains and, consequently, with high circulating levels of the Lipoprotein(a) [77]. Despite the Lipoprotein(a) measurement is always the recommended choice, a NGS panel should also include the analysis of the above polymorphisms.

Finally, exome and whole-genome sequencing should be restricted to gene discovery, due to the complexity of data analysis. However, precision medicine by deep genetic analysis helps improving the diagnosis and the management of patients. An application of genome sequencing in relation to lipid traits was recently published highlighting that this approach can identify new causative loci, new variants in gene-regions usually not analyzed and can be even used to evaluate PRS [86].

### Pathogenicity Evaluation

To date, molecular screening and identification of genetic variants is no more a challenge since many genes can be reliably analyzed by NGS, whereas the correct interpretation of the pathological role of variants remains the most difficult issue.

Considering the molecular mechanisms of FH, it appears clear that genetic variants in *APOB* and *PCSK9* should be carefully evaluated for FH pathogenicity because they can be causative of two opposite phenotypes, but their evaluation is essential also for variants in the *LDLR* gene. According to the American College of Medical Genetics and Genomics (ACMG) guidelines [87], all genetic variants should be evaluated on the basis of several aspects including, among the others, the expected variant effect, its frequency in the general population, co-segregation of variant and disease within families, the identification of several unrelated patients carrying the variant and a functional demonstration of protein alteration. Based on these criteria, variants can be classified as pathogenic, likely pathogenic, benign, likely benign or variants of uncertain significance (VUS) if the available evidence does not allow for any clear classification. Since these are general guidelines, large autonomy of evaluation is left to establish the feasibility and, in some instances, the relevance of each criterium. Given the high prevalence of the disease and the increasing interest in performing a genetic diagnosis of FH, the ACMG guidelines were improved with specifications for FH-causative genes [88,89]. Considering the molecular features of FH, the ClinGen Familial Hypercholesterolemia Variant Curation Expert Panel created specific guidelines for variants in the *LDLR* gene [89] and applied them to several variants that are now reported in the ClinVar database (https://www.ncbi.nlm.nih.gov/clinvar/ accessed on 29 December 2022) as “Reviewed by expert panel” (https://www.ncbi.nlm.nih.gov/clinvar/submitters/508055/ accessed on 29 December 2022).

These guidelines have the great advantage to reduce a personalized interpretation of pathogenicity and benignity criteria providing precise indications for the interpretation of each criterion. As an example, based on the specific number of unrelated patients with the variant, a specific strength is given to the corresponding criterion. In this way a universal interpretation of results can be made avoiding different pathogenicity evaluations from different laboratories.

Unfortunately, some data useful for pathogenicity evaluation may be available only within a single center, such as the above-mentioned criterion related with the number of unrelated patients carrying the variant or data about co-segregation of variant and disease. To date, these data are still unreported in public databases, such as ClinVar (https://www.ncbi.nlm.nih.gov/clinvar/ accessed on 29 December 2022), and cannot be used for a general interpretation of the variant. It would be very useful if all research groups or laboratories working on FH molecular diagnosis made these data publicly available for the community. Table 2 reports some available on-line resource helping to retrieve information useful for pathogenicity assessment of FH-related variants. To date, the most of relevant source of data about pathogenicity of genetic variants is the published literature.

Functional evaluation greatly contribute to the pathogenicity evaluation of variants potentially causative of FH [90], although only a few variants were tested so that the most of *APOB* and *PCSK9* variants are classified as VUS [91]. On the other hand, due to the wide expression of LDLR, patients’ cells can be easily used to test new genetic variants [92,93]. This is particularly due to the peculiar molecular mechanisms of pathogenicity, which is not based on a total defective function, but on a partial LOF and a GOF, respectively. This characteristic makes it necessary to integrate genetic analysis with a functional assay to correctly claim the pathogenic role of the variant. We experienced that some variants in *PCSK9* and in *LDLR,* which are not relevant for protein function modification [94,95].

Functional tests or predictive models could greatly contribute to the pathogenicity assessment, so that a platform for variant testing [96] and pathogenicity prediction tools based on machine learning [97] were recently implemented. New computational methods are promising tools for analyzing complex biological data [98].

## 4. Conclusions

In summary, FH was traditionally considered a monogenic disorder with dominant inheritance, but on the light of new evidence, it should be better defined as a genetic disorder with co-dominant inheritance characterized by genetic heterogeneity with an allele dosage effect and multiple modifier factors impacting phenotype and inducing a variable expression of the disease. This review summarizes the different molecular alterations identified in patients with a clinical suspect of FH and highlights the critical aspects of FH genetic diagnosis.

## Figures and Tables

**Table 1:** Genetic status and different molecular alterations identified in the different forms of familial hypercholesterolemia.

Hypercholesterolemia Form	Genes	Genetic Status
Homozygous FH	*LDLR*, *APOB*, *PCSK9*, *LDLRAP1*	at homozygous status
		at compound heterozygous status
		at double heterozygous status
Oligogenic FH	*LDLR*, *APOB*, *PCSK9*	at heterozygous status
	and	
	*ABCG5*, *ABCG8* or other modifier genes	at heterozygous status
Heterozygous FH	*LDLR*, *APOB*, *PCSK9*, *APOE* genes	heterozygous status
Polygenic hypercholesterolemia	Multiple	combination of heterozygous and homozygous variant according to determined score attribution

**Table 2:** Online resources useful to retrieve data about genetic variants.

Resource	Available Information	Website Link
ClinVar	Previous identification of variants and related condition; number of patients with the variant; pathogenicity evaluation and related evidence; literature references	https://www.ncbi.nlm.nih.gov/clinvar/ (accessed on 29 December 2022)
ClinGen curated variants in ClinVar	FH-related variants curated by the ClinGen Familial Hypercholesterolemia Variant Curation Expert Panel	https://www.ncbi.nlm.nih.gov/clinvar/submitters/508055/ (accessed on 29 December 2022)
ClinGen Familial Hypercholesterolemia Variant Curation Expert Panel	Revised pathogenicity criteria for variants in FH-causative variants; Evidence Repository about analyzed variants	https://www.clinicalgenome.org/affiliation/50004 (accessed on 29 December 2022)
Human Gene Mutation Database (HGMD)	Database of variants associated with different diseases; literature references; bioinformatic predictions	https://www.hgmd.cf.ac.uk/ac/index.php (accessed on 29 December 2022)
LOVD 3.0	Database of variants associated with different diseases; literature references; functional data; bioinformatic predictions	https://www.lovd.nl/3.0/home (accessed on 29 December 2022)
LitVar	Search instrument to retrieve information from scientific literature	https://www.ncbi.nlm.nih.gov/CBBresearch/Lu/Demo/LitVar/#!?query= (accessed on 29 December 2022)
